# Offensive Breath from Artificial Dentures

**Published:** 1874-10

**Authors:** D. C. Hawxhurst


### OFFENSIVE BREATH FROM ARTIFICIAL DENTURES.

BY D. C. HAWXHURST.

In my paper on "Offensive Breath," published in the DENTAL REGISTER, for March, 1873, I purposely omitted to mention the influence of the neglected dental plate in rendering the breath foul; that topic forms the burden of the present paper. I shall discuss the liability to overlook offensive breath arising from this, also some of the causes which occasion it, and shall point out the treatment which I have found to be the most effectual in preventing or removing it.

None but the fetid odors of neglected natural teeth can exceed in offensiveness the emanations from an uncleansed artificial denture. Its odor varies somewhat with the nature of the material out of which the plate is constructed, but much more on account of difference of physiological condition in the various secretions of the mouth. And these are important circumstances as compared with the manner and amount of cleansing to which a plate is daily subjected.

Dental plates, made of any of the materials in common use and worn in most states of health or disease, may be kept quite odorless by some very simple precautions which I shall describe.

Among cleansing agents, the first and most important is the tooth brush and pure water. To these should be added that searching friction which is best secured by the use of a good tooth powder. Without powder the brush will slip along over all but the coarsest impurities, and will leave a quantity of fine organic deposit which is even more likely to undergo putrefactive change than the coarser particles of food. There is often a little mucus and pulpy organic matter combined with earthy salts from the saliva spread over certain parts of a plate, which the brush will not readily take hold of. Through the aid of a powder the bristles are enabled to cut their way through this troublesome deposit, and render the surface of the plate free from any odor producing substance. Thorough polishing and rinsing will alone do much toward purifying a dental plate.

But if unpleasant emanations still proceed from it, the denture may be soaked in lime water and subjected to further brushing. The solution of oxide of calcium or lime water is so simple and so certain to be at hand in the dental office, that it deserves to be mentioned even though it is effectual only in a proportion of cases.

One of the best agents for disinfecting a denture to which a slight taint obstinately adheres, is permanganate of potash. A few crystals should be disolved in water enough to cover the plate. This drug is in itself unpleasant and should be well rinsed off before placing in the mouth. To be really elegant in fitting up a patient with facilities for rendering her plate tolerable and agreeable, cinnamon, orange or rose waters should be provided to be used on the plate after disinfection is accomplished. If for himself the practitioner had better be content with having made his plate oderless, anything added after this is accomplished, is liable to prove offensive to the patient, he can never be certain that the finest toilet waters will not offend somebody's delicate senses.

It is not impossible that chloride of lime may be found desirable in some obstinate cases of foul plates. A teaspoonful stirred in a glass of acidulated water furnishes a convenient and efficient mode of applying it. It is, however, a harsh disinfectant and will seldom prove agreeable.

Soap thoroughly applied with a little powder will sometimes answer every purpose of cleanliness. The necessities of the case should always be consulted and the cleansing process continued until complete purification is accomplished. Soap alone is not very efficient because of its lubricating properties. It causes the brush to glide smoothly over impurities instead of removing them.

The agents and methods of cleansing an artificial denture will vary greatly with different persons. That treatment which gives the best results where the oral fluids are normal, will often fail in presence of vitiated secretions. If there is much mucus or if the saliva is acid, lime water or some alkaline disinfectant will be found most useful, but in other cases nothing will prove so effectual as an acid agent. If there is a marked tendency to early and rapid putrefactive decomposition of the oral fluids and the alimentory atoms floating in them or lodged about the denture or mucus membrane, antiseptics may be used both as a mouth wash and plate wash. Of course the latter will be applied only after the most thorough purification and will be designed as arresters or preventers of putrefactive action. The cleansing process may thus consist of two parts, the destruction of foul odors already formed and the prevention of that retrogressive chemical action which forms them.

It is worthy of record that the essential oils may be used as agents of disinfection if nothing else is at hand. Take oil of lemon, cut with alcohol, dilute with warm water, and place in a glass; suspending the denture over the surface of the liquid after having cleansed it as well as possible by other means. I have used oil of lemon here as a type. Any of the oils or essences may furnish material or even cologne. The oil forms ozone by contact with oxygen and this is the disinfecting agent.

In the absence of all things else, pure water will soak and dissolve out impurities far more efficiently than might be supposed, if only time enough be allowed.

Alternate washings with alcohol and water constitute a most efficient mode of cleansing. The affinity between water and alcohol is very strong, being applied alternately each withdraws the other from the deepest fissures and most confined interstices of the plate, bringing out the easily soluble smell producing agents. Previous to cleansing by the water and alcohol method, the plate should have a preparatory cleansing with brush, powder and water.

In using agents which are simply frictional in their action sufficient force must be applied to carry off the deposited impurity. In applying disinfectants, designed to decompose the offensive odor or odor producing substance, sufficient time must elapse for the necessary chemical reaction to take place.

Different methods of cleansing will prove most efficient in different cases; each wearer of the artificial denture may soon learn what treatment will secure the best results in his own case. He should resort to the most searching tests of the success of whatever methods he does adopt for there is probably no other one thing outside of a slaughter house and reasonably distant from the city sewers, that is more disgusting to a patient than her dentist's fetid breath.

In a former number of this journal I have said what seemed to me to be necessary as regards oral causes of bad breath not due to artificial dentures, but I can not refrain from offering a single hint on that topic now.

It will often occur that the practitioner will thoroughly cleanse and purify his plate, but will find his breath still tainted; will still give the rubber plate smell in his breath. This is due to the impurities which belong to both plate and mucus membrane. Lying between the plate and the palate on which it rests, and to some extent overspreading the mucus membrane of the mouth or even confined between and about the natural teeth (if any remain), these impurities are quite as certain to vitiate the breath as if the denture worn had not been cleansed at all. There will often appear a thin film of semi-transparent matter on the mucus membrane, not to be seen without close scrutiny, and yet capable of giving off offensive gases; and oftentimes nothing will remove this so well as ths brush carried thoroughly over every part of the lining membrane of the mouth and over all the surfaces of the natural teeth, if any remain. The same purifying agents recommended for dental plates may be used on the natural teeth, with the exception of those of an acid nature. At all events the whole mouth must be purified, else cleansing of the plate will prove only a partial remedy, and the breath will still continue to be offensive even though the plate may have been thoroughly dealt with.

